# Improving Provision of Preanesthetic Information Through Use of the Digital Conversational Agent “MyAnesth”: Prospective Observational Trial

**DOI:** 10.2196/20455

**Published:** 2020-12-04

**Authors:** Fabrice Ferré, Nicolas Boeschlin, Bruno Bastiani, Adeline Castel, Anne Ferrier, Laetitia Bosch, Fabrice Muscari, Matt Kurrek, Olivier Fourcade, Antoine Piau, Vincent Minville

**Affiliations:** 1 Département d’Anesthésie-Réanimation Hopital Pierre-Paul Riquet CHU Purpan Toulouse France; 2 Unité Mixte de Recherche Éducation, Formation, Travail, Savoirs (UMR EFTS) Université Toulouse Jean Jaurès Toulouse France; 3 Département de chirurgie digestive et transplantation d’organes CHU Rangueil Toulouse France; 4 Department of Anesthesia University of Toronto Toronto, ON Canada; 5 Département de Gériatrie CHU Rangueil Toulouse France

**Keywords:** chatbot, digital conversational agent, preanesthetic consultation, Abric method, eHealth, digital health, anesthesia

## Abstract

**Background:**

Due to time limitations, the preanesthetic consultation (PAC) is not the best time for patients to integrate information specific to their perioperative care pathway.

**Objective:**

The main objectives of this study were to evaluate the effectiveness of a digital companion on patients' knowledge of anesthesia and their satisfaction after real-life implementation.

**Methods:**

We conducted a prospective, monocentric, comparative study using a before-and-after design. In phase 1, a 9-item self-reported anesthesia knowledge test (Delphi method) was administered to patients before and after their PAC (control group: PAC group). In phase 2, the study was repeated immediately after the implementation of a digital conversational agent, MyAnesth (@+PAC group). Patients’ satisfaction and their representations for anesthesia were also assessed using a Likert scale and the Abric method of hierarchized evocation.

**Results:**

A total of 600 tests were distributed; 205 patients and 98 patients were included in the PAC group and @+PAC group, respectively. Demographic characteristics and mean scores on the 9-point preinformation test (PAC group: 4.2 points, 95% CI 3.9-4.4; @+PAC: 4.3 points, 95% CI 4-4.7; *P*=.37) were similar in the two groups. The mean score after receiving information was better in the @+PAC group than in the PAC group (6.1 points, 95% CI 5.8-6.4 points versus 5.2 points, 95% CI 5.0-5.4 points, respectively; *P*<.001), with an added value of 0.7 points (95% CI 0.3-1.1; *P*<.001). Among the respondents in the @+PAC group, 82% found the information to be clear and appropriate, and 74% found it easily accessible. Before receiving information, the central core of patients’ representations for anesthesia was focused on the fear of being put to sleep and thereafter on caregiver skills and comfort.

**Conclusions:**

The implementation of our digital conversational agent in addition to the PAC improved patients' knowledge about their perioperative care pathway. This innovative audiovisual support seemed clear, adapted, easily accessible, and reassuring. Future studies should focus on adapting both the content and delivery of a digital conversational agent for the PAC in order to maximize its benefit to patients.

## Introduction

Currently, in France, a patient requiring scheduled surgery must go through several mandatory steps: a consultation with the surgeon, a consultation with the nurse, a preanesthetic consultation (PAC), and a preanesthetic history and physical examination.

The PAC became compulsory in France on December 5, 1994, by Decree No. 94-1050, which stated that the consultation must be “led by an anesthetist physician” who sets out an anesthesia protocol [1]. In 2018, anesthetists at University Hospital Center of Toulouse (Purpan Hospital, Toulouse, France) performed nearly 12,000 PACs, including 4500 for elective orthopedic surgery (representing over 1500 hours per year devoted to PACs in this unit).

Because only 15 to 20 minutes can be devoted to the PAC per patient, only a few minutes are dedicated to the explanation of the anesthetic (its advantages and disadvantages, and risks and alternatives, if they exist), as well as of the risks inherent in their conditions and the possible ways to reduce them [2]. The amount of new information the patient must process appears disproportionately large when compared with the short duration of the consultation. Moreover, the context of a consultation is a source of anxiety (eg, “white coat effect”) and can thus prove deleterious to the retention of such information.

At the same time, the multiplicity of tasks incumbent on anesthetists reduces the time available, which may explain why the time devoted to presenting patients with information during PACs is often reduced [3]. Providing information tailored to each patient and each situation and ensuring that it is well understood is a daily challenge. In addition, the patient’s knowledge about the anesthesia often appears limited. For instance, a 1994 study by Swinhoe and Groves [4] showed that 35% of the patients did not know that the anesthetist was a physician.

Recently, digital conversational agents (also known as chatbots) have been emerging in the health care field, including in the management of complex older populations [5]. These digital companions are very useful for communicating with the patient before or after care without overloading the clinicians. They have the benefit of being available at any time and can be used repeatedly, at home or elsewhere. In this setting, Bibault et al [6] were able to demonstrate that the quality of breast cancer information delivered by a digital conversational agent was equivalent to a specialized consultation. The interest in this type of approach as compared with the unsupervised open access to information on the internet is the ability to control the content and the accuracy of the information offered.

The main objective of this study was to develop a digital companion that could help patients to prepare for their scheduled orthopedic surgery by providing them with adapted information before their PAC.

We hypothesized that the implementation of this tool, before and in addition to the PAC, would improve the quality of the information delivered in comparison with the standard practice.

## Methods

### Experimental Design

In this before-and-after study, we planned two successive phases that allowed us to define two groups.

The first phase took place before the implementation of the digital conversational agent. Patients were evaluated by the test before and immediately after the PAC. This control group was referred to as the PAC group.

During the second phase of the study, access to the digital conversational agent, or chatbot, was granted at the moment of the surgical decision and until the PAC. The evaluation by the test was conducted before access to the chatbot and immediately after the PAC. This intervention group was referred to as the @+PAC group.

### Timeline of the Study

Phase 1 (PAC group) was carried out for 3 months, from February 1, 2019, to April 30, 2019. Phase 2 (@+PAC group) was carried out for 3 months, from June 1, 2019, to August 31, 2019. The anesthetists in the PACs were not informed of this timeline.

### Population

We included patients aged 18 to 85 years who were scheduled for a PAC before elective orthopedic surgery at the University Hospital Center of Toulouse (Purpan Hospital, Toulouse, France). The patient exclusion criteria were (1) having the PAC in a different hospital, (2) the presence of a major sensory handicap (blindness or deafness) compromising the comprehension of the information, or (3) the inability to give informed consent.

During the PAC, the physician consulted the medical documents brought by the patient, questioned and examined the patient, and informed the patient of the benefits and associated risks of the anesthetic procedures. The anesthetist could ask for complementary investigations if necessary. In addition, an information booklet about anesthetic techniques was given to the patient.

### Demographic Data Collection

Age, sex, height and weight, profession, education (number of postgraduate years completed), number of previous anesthetics received, smartphone use, type of surgery, and type of hospitalization (outpatient or inpatient) were anonymously collected.

### Interventions

The digital conversational agent MyAnesth was developed in collaboration with a company creating secure health companions (BOTdesign, Toulouse, France).

Its content was developed by 6 anesthetists from the University Hospital Center of Toulouse (orthopedic surgery unit, Purpan Hospital, Toulouse, France), taking into account data from the literature on fears generated by anesthesia [7-9]. Information considered important to be delivered to patients was then the subject of a team consensus.

The wording of the informative messages, such as frequently asked questions (FAQs) and their answers, were revised by a specialist in social and human sciences at Paul Sabatier University in Toulouse, France.

Access to the conversational agent was made possible by a URL link sent to the patient and accessible from any electronic device (ie, smartphone, tablet, or computer). This digital tool complies with all French and European regulations in terms of health data security.

**Figure 1:**
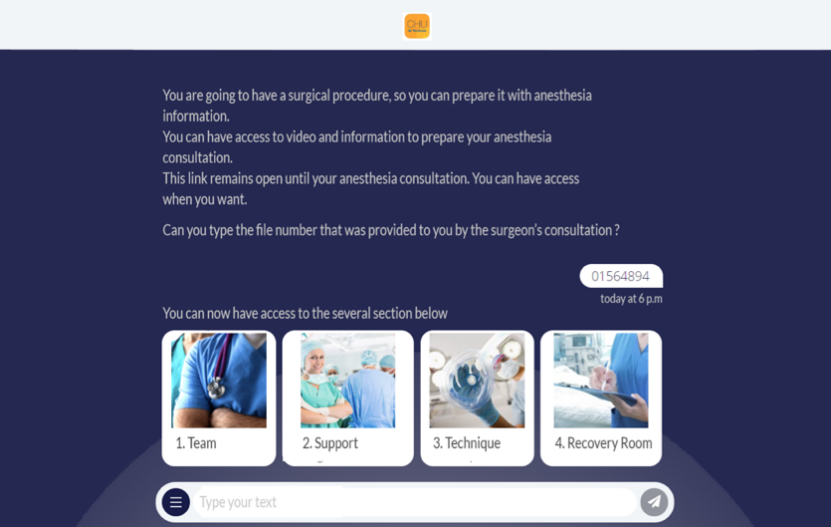
Access page to the digital conversational agent MyAnesth (BOTdesign, Toulouse, France).

Each patient could browse through 4 themed sections in the order of their choice: (1) team, (2) support, (3) technique, and (4) recovery room (Figure 1). Each section included a video, and the most FAQs and their answers, which could be accompanied by a picture (Multimedia Appendix 1). Within the support section, the content was adapted to the type of hospitalization (outpatient or inpatient), which provided the patient with more personalized information. A synthesized voice read the written information (eg, terms and conditions of use, and FAQs). The videos were subtitled in French to optimize patients’ comprehension.

A version of the digital conversational agent, for demonstration purposes only, and identical to the one used in the study, is accessible online [10].

### Primary Outcome: Self-Reported Anesthesia Knowledge Test

**Figure 2:**
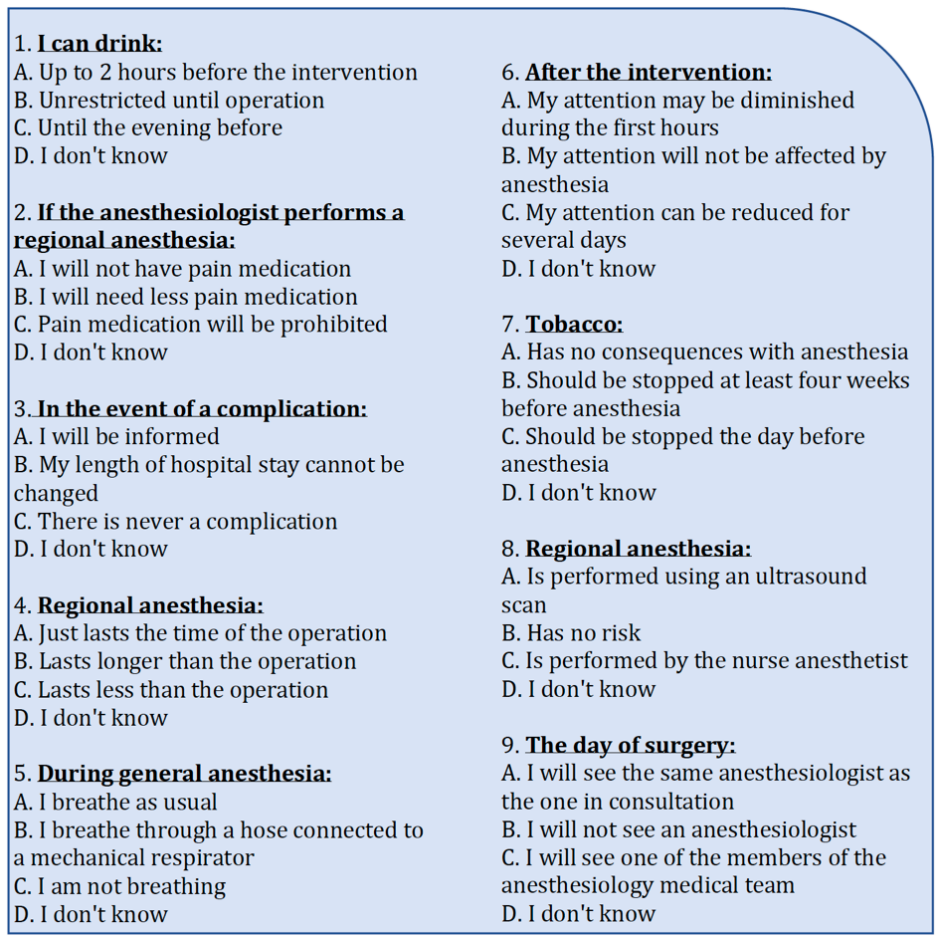
Self-reported anesthesia knowledge test. The correct answers are 1A, 2B, 3A, 4B, 5B, 6C, 7B, 8A, and 9C.

The information was considered successfully delivered if there was a significant increase in the score on the self-reported anesthesia knowledge test (Figure 2). This test consisted of 9 multiple-choice questions developed using the Delphi method [11]. The questions were simple and considered by the panel of experts as constituting the minimum amount of knowledge required before anesthesia. Each multiple-choice question had 4 answers, including the option “I don’t know.” Only one correct answer (1 point) was possible for each multiple-choice question. A wrong answer or “I don’t know” response was worth 0 points, and the total score was between 0 and 9 points.

### Secondary Outcomes

#### Patients’ Satisfaction

To evaluate patients’ satisfaction regarding the acceptability and quality of the delivered information, patients were required to answer questions rated from 1 to 5 according to the Likert method (1=strongly disagree, 2=rather disagree, 3=no opinion, 4=rather agree, and 5=strongly agree) (Multimedia Appendix 2).

#### Patients’ Representation

In order to assess the patient’s representational field for anesthesia, we used the Abric method of hierarchized evocation [12]. This method consists of asking patients a question that stimulates them to respond with 3 words or expressions associated with an inductor (word, sentence, or idea). In our case, the inductor was the word anesthesia, which was introduced by the following question: “What are the 3 words that come to your mind when you hear about anesthesia?” Patients were asked to answer this question before and after receiving information. The written order of the patient’s answers expressed the level of importance of each response (first row being the most important, third row the least important).

This method allowed us to come closer to the subject’s representations by dividing them into the core of the representation and the peripheral elements. The core constitutes “a mental filter through which reality is perceived and judged” [12]. It is rather independent from the context, contrary to the peripheral elements, which adapt the core to the diversity of the context [12].

The treatment of these data consisted of an analysis that took into account the frequencies of response of a word and of the written order of the responses [13,14]. This analysis allowed us to identify the core zone and the first periphery corresponding to the strongest frequencies in rows 1 and 2, followed by the contrasted elements zone and the second periphery corresponding to the weakest frequencies and row 3 [14].

### Sample Size Projection

No data were available from the literature to allow us to calculate a priori the sample size required to identify an increase in the anesthesia knowledge test score. In a pilot study conducted on 30 patients, we identified an increase of 1.0 point (SD 1.3 points) in test scores taken before and after the PAC (4 of 9 points and 5 of 9 points, respectively) on the anesthesia knowledge test.

Considering that the implementation of the digital companion could allow the gain of 1 more point (increase judged to be clinically relevant) compared with the PAC alone, we calculated that 48 patients per phase would be required to demonstrate this difference with a type I error of 5% and a power of 90%. Taking into account the number of patients potentially lost, the technical difficulties inherent to the use of a digital conversational agent not yet tested, and the total number of patients seen in our PACs, we planned for 2 successive periods of 3 months each to include all eligible patients.

### Statistical Analysis

The normality of the data was assessed using the Shapiro-Wilk test. The qualitative data were expressed as numbers (percentages). The quantitative data were expressed as median (range) or mean (SD) as appropriate. The categorical variables were compared using the Fisher exact test or the chi-square test. Continuous variables were compared using the Wilcoxon signed-rank test or the Student t test as appropriate. The statistical analysis was done using MedCalc Statistical Software, version 12.6.1 (MedCalc Software bvba, Ostend, Belgium). *P*<.05 was considered statistically significant.

### Ethics

The connection to the digital conversational agent was made anonymous by a 4-digit number delivered by one of the investigating physicians during the interview with the programming nurse. Even though no information about the participants’ health condition was asked at the time of the connection, the company BOTdesign (Toulouse, France) had access neither to the patients’ identity nor to their internet protocol address. This strategy of data protection was decided in agreement with the eHealth committee of the University Center Hospital of Toulouse.

This research was considered to be an experimentation in educational sciences looking to (1) evaluate the quality of the information delivered through an innovative pedagogical tool, and (2) investigate the participants’ satisfaction. Hence, this research was deemed to fall outside the Jardé law. For each patient, one of the investigating physicians delivered information about the methods of this research and ensured their nonopposition to participate. The lack of return of the questionnaire was considered a refusal to participate.

This study did not present any risk to the participants, nor did it modify the regular care process or the time require to care for the patients.

## Results

### Patients’ Characteristics

**Figure 3:**
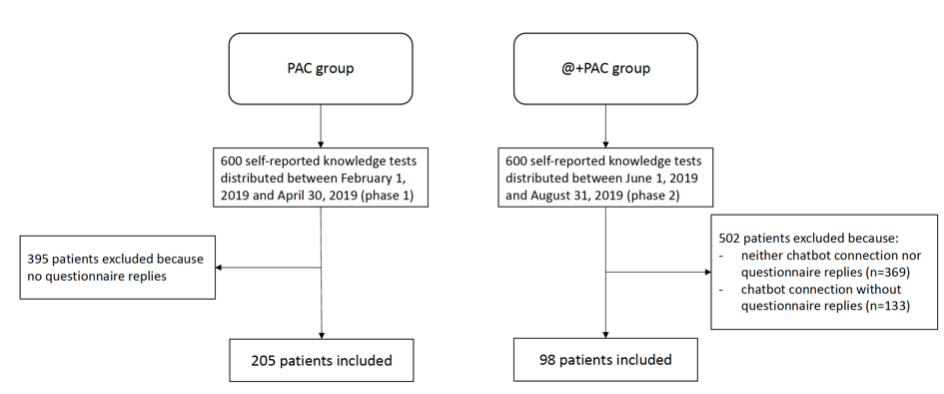
Flow chart of patient selection. PAC: preanesthetic consultation; @+PAC: digital conversational agent and preanesthetic consultation.

A total of 303 patients completed the questionnaire and were analyzed during the study period. Of these, 205 patients were included in the PAC group (phase 1) and 98 were included in the @+PAC group (phase 2). The flow chart of patient selection is presented in Figure 3.

Demographic characteristics are presented in Table 1. The number of previous anesthetics received and the number of years of postgraduate education were comparable between the groups. Most patients included in the study were outpatients. Both groups had a high rate of internet access on their smartphones or at home (Table 1).

**Table 1:** Patients’ characteristics.

	PAC^a^ group (n=205)	@+PAC^b^ group (n=98)	*P* value
Age (years), mean (range)	48 (18-85)	50 (18-74)	.73
Female sex, n (%)	95 (46.3)	41 (41.8)	.54
Number of previous anesthetics received, mean (SD)	4 (3.7)	3.9 (2.8)	.79
Number of postgraduate years of education, mean (SD)	1.4 (1.9)	1.5 (1.9)	.70
Internet access at home, n (%)	187 (91.2)	91 (92.9)	.64
Internet access with smartphone, n (%)	174 (84.9)	86 (87.8)	.60
Outpatient, n (%)	137 (66.8)	71 (72.4)	.22

^a^PAC: preanesthetic consultation.

^b^@+PAC: digital conversational agent and preanesthetic consultation.

### Anesthesia Knowledge Test

**Figure 4:**
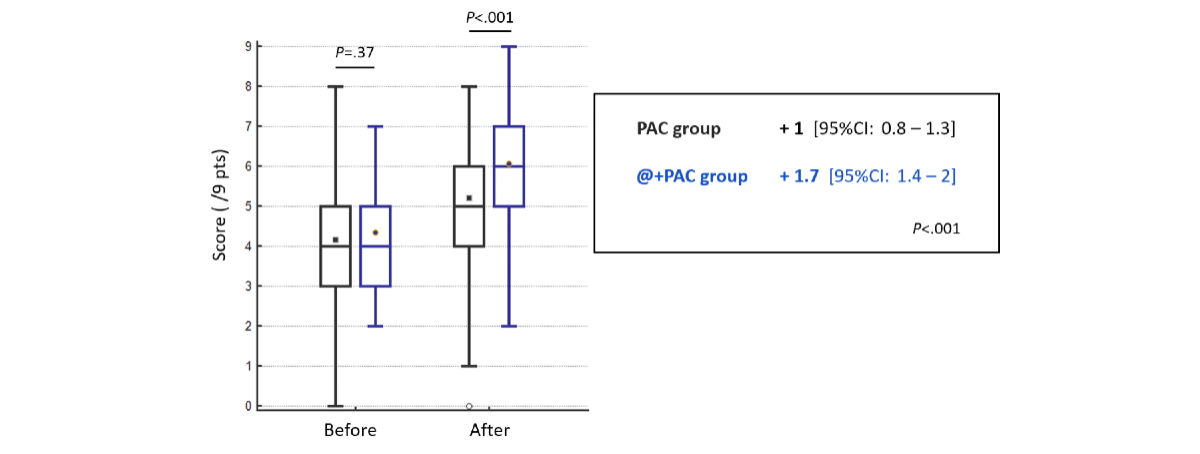
Comparison of anesthesia knowledge test scores between patient groups. Means are shown as markers within the box of 25th and 75th percentile values; whiskers represent ranges. PAC: preanesthetic consultation; @+PAC: digital conversational agent and preanesthetic consultation; pts: points.

The results of the anesthesia knowledge test are shown in Table 2 and illustrated in Figure 4. The implementation of the digital conversational agent led to an increase in the test score by 0.7 points (95% CI 0.3-1.1; *P*<.001).

**Table 2:** Anesthesia knowledge test results.

	PAC^a^ group (n=205), mean (95% CI)	@+PAC^b^ group (n=98), mean (95% CI)	*P* value
Knowledge test score^c^ before receiving information	4.2 (3.9-4.4)	4.3 (4.0-4.7)	.37
Knowledge test score^a^ after receiving information	5.2 (5.0-5.4)	6.1 (5.8-6.4)	<.001
Score improvement	+1.0 (0.8-1.3)	+1.7 (1.4-2.0)	<.001

^a^PAC: preanesthetic consultation

^b^@+PAC: digital conversational agent and preanesthetic consultation

^c^Score range is 0-9 points.

### Patients’ Satisfaction

Among patients in the @+PAC group, 74% (73/98) agreed that the digital conversational agent was easy to access, and 82% (80/98) found the information to be clear and appropriate (Table 3).

In the PAC group, 34.1% (70/205) of patients agreed and 54.1% (111/205) disagreed with the following statement: “I wish I had received information before the anesthetic consultation.”

**Table 3:** Digital conversational agent users’ satisfaction analysis (n=98).

	Digital conversational agent accessibility, n (%)	Digital conversational agent content quality, n (%)
Number of respondents	98 (100)	98 (100)
Number of respondents who rated 1-2^a^	10 (10)	4 (4)
Number of respondents who rated 3^a^	15 (15)	14 (14)
Number of respondents who rated 4-5^a^	73 (74)	80 (82)

^a^Likert scale: 1=strongly disagree, 2=rather disagree, 3=no opinion, 4=rather agree, 5=strongly agree.

Among patients in the @+PAC group, the videos in the support and technique sections of the digital conversational agent were the most watched among viewers (88/98, 90%). The video in the section about the recovery room was the least popular, but was still watched by 68% (67/98) of the patients who logged in.

The apparent success of the videos contrasts with the low consultation rate regarding information delivered in the FAQs. Indeed, among the patients in the @+PAC group, only 4% (4/98) consulted all 11 FAQs, and 66% (65/98) did not consult any.

### Patients’ Representations

Responses from both groups were pooled.

**Figure 5:**
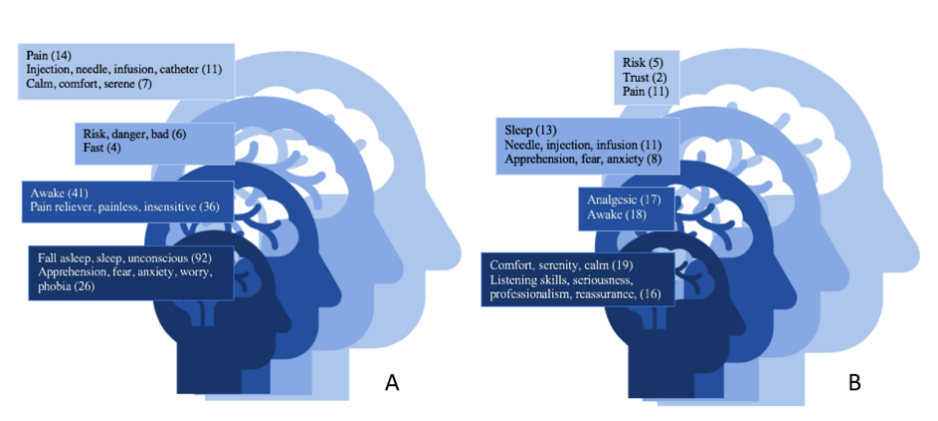
Graphic representation of patients' feelings before (A) and after (B) receiving information using the Abric method of hierarchized evocation. From the center to the periphery are spread out: the central core area, the first periphery, the contrasting elements, and the second periphery. The number of occurrences of the terms is indicated in parentheses.

Before receiving the preanesthetic information, the 233 patient responses indicated a core representation of anesthesia made up of concerns related to sedation (92 occurrences in the first row) and to apprehension (26 occurrences in the first row) (Figure 5A). This apprehension was confirmed by the elements from the first periphery, with the notion of awakening in particular (41 occurrences in the second row) associated with the problem of pain expectations (36 occurrences in the second row).

The contrasted elements were risk and fasting, brought up in row 1, although rarely. Infrequently brought up in lower rows, pain (14 occurrences), injection (11 occurrences), and calm (7 occurrences) defined the second periphery.

After information was received, the 149 responses showed a change in the core representation, with a new interest in comfort (19 occurrences in the first row) and skills of the health care professionals (16 occurrences in the first row) (Figure 5B). Elements related to the absence of pain and the awakening no longer presented the initial strength, with 17 and 18 occurrences, respectively, in the third rows. However, elements related to injections (11 occurrences) came to the first row even if the frequency was still low; elements regarding apprehension remained in the first row but became rarer (8 occurrences). Risk, which was previously in the first row, moved to the third row and at a low frequency (5 occurrences). The notion of trust also appeared (2 occurrences in the second row).

## Discussion

Using a digital conversational agent before the PAC lead to a significant improvement in the patients’ knowledge of anesthesia. The high rate of internet access in addition to the high acceptability of the digital conversational agent encourage us to widely develop this tool. Our results highlight the lack of patients’ knowledge about anesthesia. Indeed, no patient in either group received a passing score on the test before information delivery. In addition, the low increase in test scores (by 1 point) after PAC alone speaks in favor of identifying the best platforms and methods for delivering quality information.

Patient education through the use of information and communication technologies is a rapidly developing field that promises to improve patient outcomes while simultaneously using fewer human resources. In terms of content format, our study found that videos were consulted much more than text (ie, FAQs). Our results are in accordance with the meta-analysis conducted by Lee et al [15] in which patients being offered explanatory videos had better odds of correctly answering questions regarding anesthesia (relative risk 6.6, 95% CI 2.1-21.5). Hering et al [16] also showed an improvement in patients’ satisfaction and knowledge by visiting a website before the PAC.

In our study, the most-viewed videos were related to anesthesia techniques and hospitalization modalities. Of course, these results could guide us toward themes that patients may wish to address before surgery.

Managing the content and accuracy of information offered enables better guidance with patients’ online research, which may be beneficial because unsupervised online research can occasionally create anxiety [17].

Another advantage of digital tools is monitoring and re-evaluation. The data from the connections to the digital companion enable us to reliably monitor its use, allowing us to regularly readjust and update the content, unlike paper-based materials, where use cannot be evaluated.

Our results confirm that increasing patients’ information is necessary to improve their satisfaction [18-21] and knowledge [16,19], as well as to reduce their anxiety [22-25].

We made the choice to place the interaction with the digital companion before the PAC to stimulate patients’ curiosity and prompt them to ask themselves questions about the modes of anesthesia and their care pathways. However, surprisingly, we noticed during phase 1 that almost one-half of the patients did not wish to receive any information before the PAC. Thus, the question of information timing remains open. Interestingly, the use of a digital companion is a real advantage because it is easily accessible at any time, before and after consultation. In addition, this kind of interactive online tool could make communication with patients more efficient, especially when hospitals are not accessible, such as during a pandemic.

Giving information before a consultation could also shorten the length of the PAC without impacting patients’ satisfaction [26,27]. Taylor et al [27] evaluated patients’ completion of a numerical questionnaire before consulting with the anesthetist nurse. The mean consultation time of the group being offered the questionnaire was 12 minutes compared with 27 minutes for the group who was not offered the questionnaire (*P*<.001).

Other studies have shown the possibility of reliably automating the score calculation of patients’ anesthetic risks [28]. Using a digital questionnaire before PAC would reduce the consultation duration while insuring a high level of quality and patient satisfaction [29].

For the first time, the method of hierarchized evocation was used to observe patients’ feelings toward anesthesia. Overall, our results display patients’ anxiety before receiving information and the benefits of getting information on how anesthesia is perceived. The terms skill, listening, seriousness, professionalism, comfort, and reassurance frequently appear after information is received. The semantic field category of these words is the correlate of a low preoperative anxiety level.

Our study presents several limits. First, the results were obtained by enrolling patients scheduled for elective surgery, and the results may not be able to be extrapolated to emergency cases where the consultation with a digital tool may not be feasible. Second, only 16.3% (98/600) of questionnaires distributed during phase 2 were completed. Characteristics of the nonresponders (eg, advanced age) could be very informative in identifying possible barriers. Finally, we have not evaluated physician satisfaction, which could help to identify the best perioperative health education support for the future.

To conclude, we have shown improvement in patients’ knowledge about their care pathway when a digital conversational agent was used before the PAC. Despite the encouraging results, the overall uptake of the tool was relatively low and, even when used, did not achieve maximum impact. Future studies should focus on adapting both the content and the delivery of a digital conversational agent for the PAC in order to maximize its benefit to patients.

## Appendix

Multimedia Appendix 1 Examples of Frequently Asked Questions extracted from the chatbot.

Multimedia Appendix 2 Satisfaction questions assessed by the Likert scale.

## Abbreviations

PAC: preanesthetic consultation
