# Supplementary material for: Improving Provision of Preanesthetic Information Through Use of the Digital Conversational Agent “MyAnesth”: Prospective Observational Trial
Source: J Med Internet Res. 2020 Dec 4;22(12):e20455. doi: 10.2196/20455 (PMC7748965; doi:10.2196/20455)
Supplement: Multimedia Appendix 1 [file jmir_v22i12e20455_app1.docx]

**Appendix 1. Examples of Frequently Asked Questions extracted from the chatbot:**

- “Team” theme
- Do I need a blood group card before surgery?

We don't need to know your blood type to perform anesthesia. The anesthesiologist will decide to prescribe a blood type determination if he deems it necessary based on the type of surgery. If you have a card, bring it on the day of the preanesthetic consultation to ensure its validity.

- Do I need to wash myself with povidone iodée (Betadine®) before the surgery?

We ask you to wash with a new soap and shampoo the day before and the morning of the intervention.

- Will there be nursing or physiotherapy after the operation?

Each patient and each intervention are singular; your surgeon will prescribe and explain the most adapted care to your postoperative period.

- “Support” theme

. Outpatient hospitalization

- Can I stay overnight alone after the procedure?

The decision will be made during the consultation depending on your state of health, the type of intervention and anesthesia and the proximity of your accommodation.

- Who can sign me the transport voucher or the work stoppage?

The surgeon is the prescriber of your surgery. In order for your rights to be respected, the documents must be signed by his team.

. Traditional hospitalization

- How long will I stay in hospital?

Each patient recovers at his own pace from an intervention. We will therefore suggest that you be hospitalized as long as it is necessary and beneficial for your care.

- Can my loved ones accompany me?

Relatives can of course accompany you by respecting the visiting hours. Please ask the nurses in the service.

- “Technique” theme
- I would like to see what a theatre looks like.


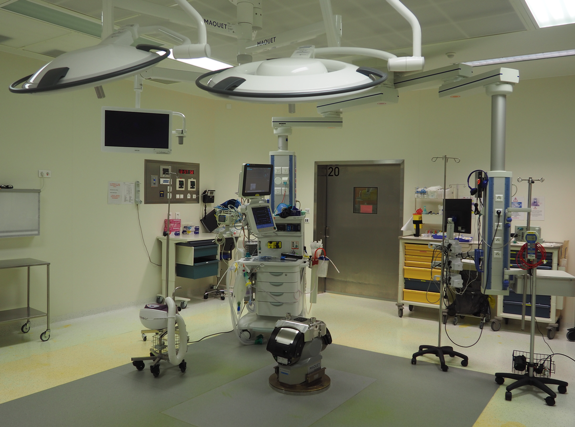


Here is a theatre at Purpan Hospital.

- What does regional anesthesia look like?


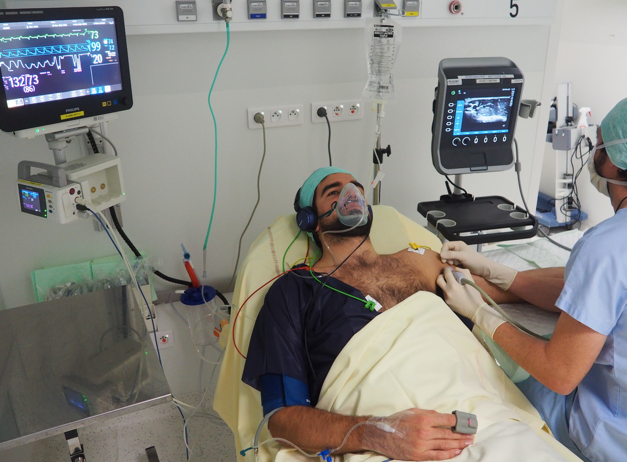


Here is a photo of an anesthesiologist performing a nerve block of the upper limb. The patient can listen to music or chat with the team during the procedure. It lasts several minutes.

- Can I not wake up after a general anesthetic?

Anesthesia drugs are eliminated by your body, which inevitably leads to your waking up. The anesthesia consultation makes it possible to assess the risks and plan examinations to find out your state of health. The monitoring tools make it possible to detect failures and treat them with all the appropriate techniques available at the hospital.

- Is there a risk of paralysis with regional anesthesia?

Nerve damage is rare. Before performing anesthesia, we ensure that there are no contraindications. Sensitivity disturbances may persist several hours or days. If you have any concerns, let your doctor know.

- Is there a way to anesthetize without an injection?

Any anesthesia requires an infusion. If this act seems unpleasant to you, the team will suggest methods to improve your comfort.
