# Supplementary material for: Improving Provision of Preanesthetic Information Through Use of the Digital Conversational Agent “MyAnesth”: Prospective Observational Trial
Source: J Med Internet Res. 2020 Dec 4;22(12):e20455. doi: 10.2196/20455 (PMC7748965; doi:10.2196/20455)
Supplement: Multimedia Appendix 2 [file jmir_v22i12e20455_app2.docx]

**Appendix 2. Satisfaction questions assessed by the Likert scale:**

To find out how satisfied you are with the quality of the information, please rate the following options on a scale of 1 to 5: (1 = strongly disagree, 2 = rather disagree, 3 = no opinion, 4 = rather agree, 5 = strongly agree).

- PAC Group

I would have liked to receive information before the preanesthetic consultation.

1 2 3 4 5

- @+PAC Group

Access to the digital conversational agent tool was easy

1 2 3 4 5

The information delivered by the digital conversational agent was clear and adapted

1 2 3 4 5
